# Disclosure of onset-predictive biomarker results to research participants at risk of genetic frontotemporal dementia: a European perspective

**DOI:** 10.1186/s13195-025-01930-4

**Published:** 2025-12-13

**Authors:** Charlotte H. Graafland, Eline M. Bunnik, Barbara Borroni, Arabella Bouzigues, Sergi Borrego-Écija, Eino Solje, Caroline Graff, Jonathan D. Rohrer, John C. van Swieten, Laura Donker Kaat, Harro Seelaar

**Affiliations:** 1 Department of Public Health, Program of Medical Ethics, Philosophy and History of Medicine, Erasmus University Medical Center, Rotterdam, The Netherlands; 2 Department of Clinical and Experimental Sciences, University of Brescia, Brescia, Italy; 3 Molecular Markers Laboratory, IRCCS Istituto Centro San Giovanni di Dio, Fatebenefratelli, Brescia, Italy; 4 Dementia Research Centre, Department of Neurodegenerative Disease, UCL Queen Square Institute of Neurology, London, UK; 5 Alzheimer’s Disease and Other Cognitive Disorders Unit, Neurology Department, Hospital Clínic-Institut d’Investigacions Biomediques August Pi I Sunyer (IDIBAPS), Barcelona, Spain; 6 Institute of Clinical Medicine – Neurology, University of Eastern Finland, Kuopio, Finland; 7 Neuro Center – Neurology, Kuopio University Hospital, Kuopio, Finland; 8 Department NVS, Centre for Alzheimer Research, Division of Neurogenetics, Karolinska Institute, Stockholm, Sweden; 9 Unit for Hereditary Dementias, Theme Aging, Karolinska University Hospital, Stockholm, Sweden; 10 Department of Neurology and Alzheimer Center, Erasmus University Medical Center, Rotterdam, The Netherlands; 11 Department of Clinical Genetics, Erasmus University Medical Center, Rotterdam, The Netherlands

**Keywords:** Clinical trials, Disclosure, Frontotemporal dementia, Ethics, Biomarkers

## Abstract

**Background:**

As understanding of biomarkers for genetic frontotemporal dementia (FTD) advances, there is a need to develop onset-predictive biomarker tests (OPBTs) to detect changes before the onset of symptoms. OPBTs can be used to recruit carriers or individuals at 50% risk of carriership into clinical trials of investigational therapies targeting the preclinical and prodromal phases of FTD. OPBT results should be disclosed as part of the informed consent process, with positive results indicating that symptom onset is likely in the next few years. This information can be psychologically burdensome, especially in individuals at 50% risk, for whom a positive OPBT result would reveal their genetic status. There is a need for ethical guidance for disclosure processes to help researchers implement disclosure of OPBT results responsibly at their study sites.

**Methods:**

Existing literature on disclosure of genetic and biomarker results in neurodegenerative conditions informed the design of this disclosure process for OPBT in FTD. Drafts were discussed with the multidisciplinary research team, scientific and clinical FTD experts across European countries, and other stakeholders and revised accordingly.

**Results:**

The suggested disclosure process provides guidance for first-time or repeated disclosure of OPBT results to carriers or individuals at 50% risk of genetic FTD in research settings.

**Conclusions:**

Researchers involved in clinical trials using OPBTs can adopt this disclosure process as a framework for responsible communication of OPBT results at their study site. The process was designed for international applicability and facilitates the alignment of disclosure processes for clinical trial recruitment across European countries.

**Supplementary Information:**

The online version contains supplementary material available at 10.1186/s13195-025-01930-4.

## Background

Carriers of genetic mutations causing frontotemporal dementia (FTD) are at high risk of developing symptoms of FTD [1]. Depending on the genetic mutation, the disease is pathologically characterized by an accumulation of either TAR DNA-binding protein-43 (TDP-43) or tau in the brain [2]. The neurodegeneration resulting from these proteinopathies is irreversible. Therefore, therapeutic approaches for FTD have focused on compounds that interfere early in pathological pathways in order to prevent or delay neuronal injury. These investigational compounds are thought to be most effective when administered in a prodromal or even preclinical stage, when there is evidence of pathological changes but before symptoms have manifested [3]. However, in the absence of symptoms it is difficult to predict when carriers of genetic FTD are in this presumed window of opportunity, as age of onset varies from 35 to 80 even within families carrying the same mutation [4, 5]. To allow for timely recruitment into clinical trials, new “onset-predictive” biomarker tests (OPBTs) like neurofilament light chain (NfL) are being developed to detect changes in carriers of pathogenetic variants in genes causing genetic FTD a few years before the onset of symptoms [6–8].

Although current OPBTs have not been sufficiently validated for prediction of onset in individual carriers, they can be used as an inclusion criterion for clinical trials. Indeed, NfL testing has already been used as an inclusion criterion in a clinical trial for latozinemab in progranulin-related FTD [9]. A common recruitment strategy for clinical trials in FTD is to invite participants in longitudinal cohort studies, such as the Genetic Frontotemporal Initiative (GENFI) in Europe and Canada and the ARTFL-LEFFTDS Longitudinal Frontotemporal Lobar Degeneration Study (ALLFTD) in the U.S. In these cohorts, the natural history of FTD is studied in genetic carriers or those at 50% risk of genetic FTD, of whom a subset will develop first disease changes and be eligible for clinical trial participation. Over time, and if more clinical trials become available, these studies may also serve as “trial-ready cohorts” to provide easy access to potential participants for clinical trials who are regularly monitored and biomarker-tested.

The use of OPBTs to assess eligibility for clinical trial participation, however, raises ethical concerns. Clinical trials usually employ “transparent enrolment”, meaning that individuals included in the study are informed of the reasons why they are eligible. If testing positive on OPBTs is an inclusion criterion, potential participants would have to be informed that they are eligible for clinical trial participation because they are expected to develop symptoms of FTD in the coming years. This news may be psychologically burdensome, especially for individuals who have not learned their genetic status and only know they are at 50% risk, who make up 58.3% of current GENFI participants (personal communication GENFI team, 12 May 2025).

There is a dearth of guidance on how to responsibly disclose OPBT results to individuals at risk of genetic FTD who are in the preclinical or prodromal phase of the disease and could be eligible for clinical trial participation. Recently, we have discussed ethical considerations regarding whether and to whom to disclose OPBT results [10]. We conclude that OPBT can be disclosed responsibly under the condition that potential clinical trial participants, either known carriers or individuals at 50% risk of carrying genetic FTD, are offered high-quality pre-test counselling and follow-up. In this article, we present a basic framework for the responsible disclosure of OPBT results in the context of clinical trial recruitment to individuals who are in the presymptomatic, but not prodromal, stage of genetic FTD. Following definitions used in papers on predicting onset of FTD, we define the presymptomatic stage as a Clinical Dementia Rating (CDR^®^) Dementia Staging Instrument plus NACC FTLD Behavior & Language Domains (CDR^®^ plus NACC FTLD) score of 0 [3, 6, 7]. The framework was designed to be applicable to study sites across European countries and can be adapted to fit the local context of specific study sites within these countries.

## Methods

### Process development

We first examined existing literature detailing recommended processes for genetic counselling and disclosure of biomarker test results, drawing from the literature on HD [11, 12], genetic ALS [13, 14] and Alzheimer’s disease (AD) [15–21]. Some processes detailed counselling and disclosure of genetic status, either for autosomal dominant HD [11, 12] or ALS [13] or the susceptibility gene *APOE* for AD [15, 17, 19]. Other processes concentrated on biomarker disclosure, either amyloid status in AD [16, 18, 20] or NfL measures in ALS [14]. Two processes were specifically aimed at disclosure in the context of clinical trial recruitment [14, 17], and two concentrated on condensed processes of disclosure [15, 19]. For each of the included studies, we analysed the elements of the recommended processes and assessed their relevance for the disclosure of OPBT results in genetic FTD.

We then used the results of this analysis and recent qualitative findings on the perspectives of carriers and individuals at 50% risk of genetic FTD on OPBT and their preferences for disclosure [22] to construe, supplement and tailor a preliminary draft of the disclosure process for OPBT in genetic FTD. An overview of elements that were adopted from the literature is provided in Supplement 1. This preliminary draft was discussed with the local multidisciplinary FTD research team at Erasmus University Medical Center (including clinical geneticists, neurologists, neuropsychologists and ethicists) in January 2024 and with psychologists involved in genetic counselling for FTD in October 2024. Both meetings led to revisions of the first draft.

### Process refinement

Following methods for the development of other informed consent and counselling frameworks [18, 23], we presented and discussed the draft process several times with international FTD experts. We hosted two online consultation meetings in November and December 2024 with all co-authors who are principal investigators of GENFI study sites in Europe. They were sent the draft process in advance and asked to discuss agreements and disagreements and provide suggestions for improvement. The meetings were recorded and transcribed. Based on discussions and suggestions, the draft of the disclosure process was further refined.

This draft was then sent to the GENFI Participant Engagement Board (PEB) in June 2025 to gather further input. The PEB consists of participants who are either aware or unaware of their genetic status from several GENFI sites, including from the United Kingdom, Italy, Spain, Sweden and Canada. The PEB’s feedback included concerns about participants developing suicidal ideation after biomarker disclosure and the need for high-quality counselling, the importance of determining clinical trial eligibility before OPBT is disclosed, concerns about the accuracy of the test, the importance of correcting therapeutic misconceptions during counselling, the need for qualified professionals to execute the process, the need for additional training in communication for these professionals, and the need for follow-up support for participants’ family members. The disclosure process was revised according to feedback from the PEB. Finally, all FTD experts involved in the consultation meetings provided written feedback on the disclosure process until agreement on the final version was reached.

## Results

**Fig. 1:**
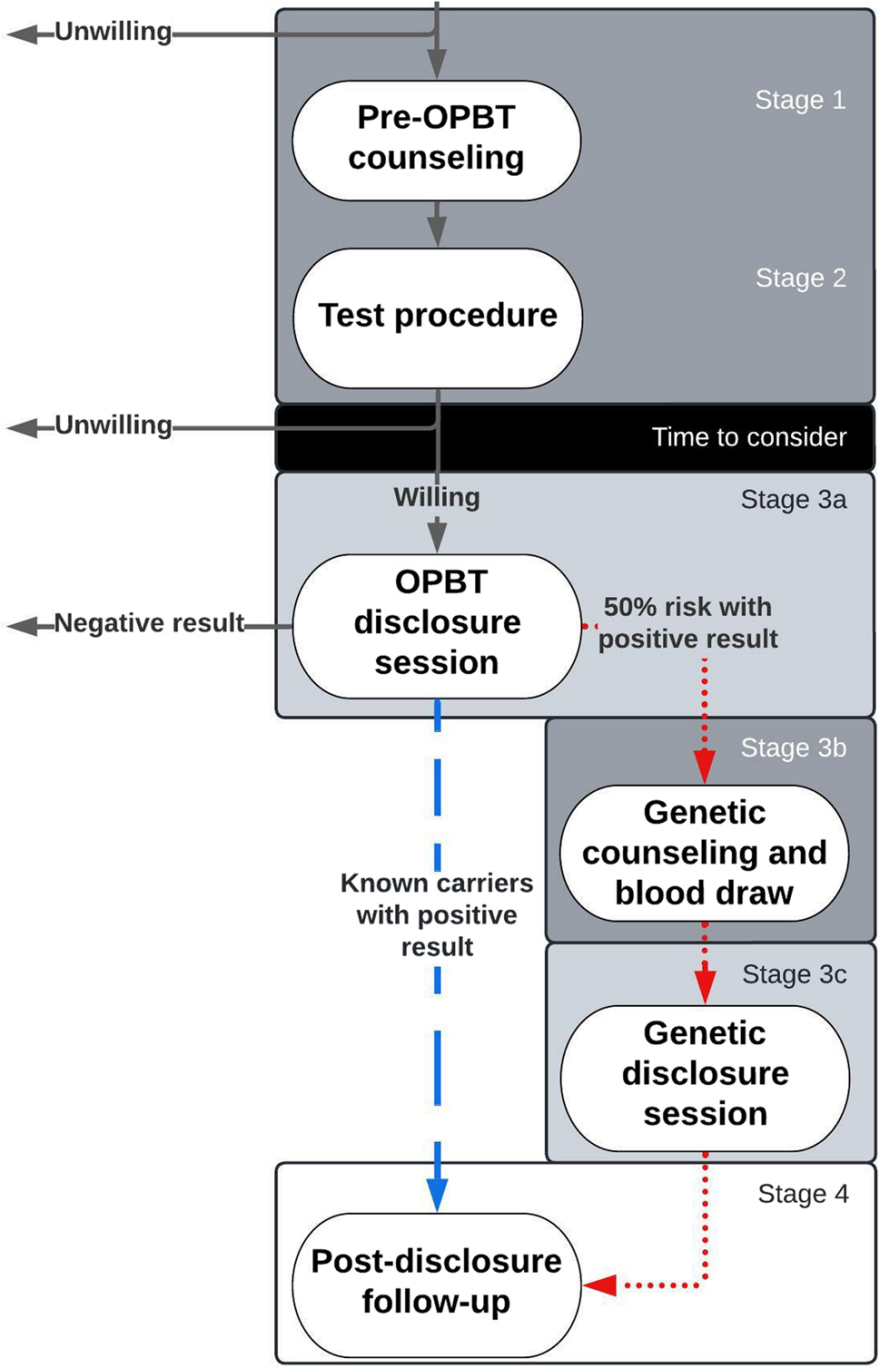
Flowchart of disclosure process for disclosure of OPBT results. Legend: Arrows: solid grey: all; dotted red: individuals at 50% risk; striped blue: known carriers. Blocks: dark grey: in-person; medium grey: in-person or videocall; white: videocall or telephone

The disclosure process for OPBT in FTD consists of two parts. The first part details a recommended process for the first time OPBT results are disclosed to research participants. However, in longitudinal cohort studies OPBT may be repeated at regular intervals as part of data collection and/or monitoring eligibility for clinical trial participation. Each repetition of the test would require renewed consent to disclose, but a full repetition of the first-time disclosure process seems disproportionately extensive and burdensome. Therefore, the second part of the process describes a condensed process for repeated disclosure of OPBT results. Both the full and the condensed process consist of four stages: pre-test counselling, test procedure, disclosure session(s) and post-disclosure follow-up (see Fig. 1). The participant is free to decline disclosure at any time during the process.

The disclosure process was designed for known carriers and individuals at 50% risk. Some counselling information and process steps are specific to individuals at 50% risk (in italics) and some to known carriers (underlined). The process was not developed for a specific OPBT and needs to be adapted to fit the features of the OPBT(s) used. The presentation of results of the OPBT will depend on the inclusion criteria of the clinical trial for which participants are screened, e.g. a ‘positive’ result (above cut-off for inclusion) or a ‘negative’ result (below cut-off for inclusion).

### Part 1: First-time disclosure of OPBT results

Any individual who may be interested in OPBT results is recommended but not obliged to go through the disclosure process. If a person declines pre-test counselling, the information explained in the pre-test counselling session should be provided in written form. Table 1 describes each of the stages in the process. Table 2 suggests discussion points for the pre-test counselling session.

Counselling should be provided by a physician who is specialized in genetic FTD and skilled at risk communication, which could be a neurologist, psychiatrist, geriatrician, genetic counsellor or clinical geneticist, depending on the local context. Individuals at 50% risk specifically should be counselled by a clinical geneticist or genetic counsellor, given the potential genetic implications of a positive OPBT result. Counsellors should be aware of the potential for serious psychological impact of OPBT disclosure, similar to genetic disclosure, for both carriers and family members. If necessary, counsellors should receive additional communication and psychological training. Ideally, the physician is already acquainted with the potential participant through previous visits and is able to tailor risk communication to the counselee’s needs. Participants are encouraged to bring a support person to the pre-test counselling and disclosure session.

The participant’s considerations regarding disclosure of OPBT results should be documented in their file to inform future pre-test counselling sessions with repeated OPBT. At the end of the pre-test counselling session, participants should be offered additional sessions with, for example, a clinical geneticist or a medical psychologist, in case they wish or need additional support to decide about OPBT. Following Largent et al. 2023 [20], participants with psychiatric symptoms should not necessarily be excluded from receiving OPBT results, especially if their symptoms are tied to their risk of neurodegenerative disease. These participants should be offered appropriate care, and the physician should use clinical judgement to determine whether to go through with OPBT. Post-disclosure follow-up is highly recommended for participants with a positive OPBT result and for their family members, but can also be offered to participants with negative OPBT results if they wish.

**Table 1:** Stages of the disclosure process for known carriers and individuals at 50% risk of genetic FTD

**Stage 1: Pre-test counselling session with FTD physician-researcher**
Discuss talking points in Table 2
Stress that OPBT is offered to assess eligibility for a clinical trial of an investigational treatment with unknown efficacy
Provide information about the possibilities for participation in a clinical trial in case of a positive result, and describe potential risks and benefits of participation in the trial
Test the individual’s understanding of the test and its implications, e.g., using a teach-back method
Discuss process and preferences for the disclosure session (planned/unplanned, in-person/videocall)
Discuss preferences for follow-up and/or clinical trial invitation (if applicable) after disclosure
Provide a written summary of relevant information and a contact phone number or email address for further questions
Offer additional sessions with a clinical geneticist, medical psychologist or other relevant medical professional, if the participant wants to discuss OPBT more extensively or with other specialists
Participants are given time to consider whether they want to receive OPBT results
**Stage 2: Test procedure**
Any material necessary for the test is collected [in longitudinal cohort studies: during the study visit]
**Stage 3a: OPBT disclosure with FTD physician-researcher [some time after pre-test counselling]**
Briefly summarize the purpose of the disclosure session and the type of test that was done
Disclose the test result using standardized language and make room for (emotional) reactions
Repeat information about the predictive value and clinical significance of the result
In case of negative OPBT result: repeat that OPBT results may come back positive in the future but that repeated testing is optional or might not be available, *and that the participant may still be a carrier*
* In case of positive OPBT result: stress the inconclusiveness of the OPBT result without a genetic test result*,* repeat the need for a genetic test to confirm carrier status*,* and discuss willingness to learn genetic result*
* In case of positive OPBT result: discuss referral to the clinical geneticist for genetic testing in the clinic*
* In case of positive OPBT result: offer to refer participants to the relevant medical professional(s) to discuss the impact of the test result*,* also on life planning*
* In case of positive OPBT result: discuss implications of the test result for participation in clinical trials*
* In case of positive OPBT result: discuss implications for family members and provide written summary for family communication*
* Provide information on post-disclosure follow-up*
***Stage 3b: Genetic counselling and collecting blood [in clinical care]***
* Revisit implications of genetic test results for the interpretation of the clinical significance of the OPBT result and opportunities for clinical trial participation*
* Revisit implications of genetic test results for family members*
* If they wish to continue with genetic testing*,* collect blood for genetic test*
* Provide information on next steps and discuss their preferences for disclosure of genetic test results*
***Stage 3c: Genetic disclosure session [in clinical care]***
* Briefly summarize the purpose of the disclosure session*
* Disclose the test result using standardized language and explain its implications for the interpretation of the OPBT result*,* making room for (emotional) reactions*
* In case of negative genetic result: explain other potential causes for positive OPBT result*
* In case of positive genetic result: discuss the implications of the genetic result for family members and life planning*,* and provide written summary for family communication*
* In case of positive genetic result: offer to refer participants to the relevant medical professional(s) to discuss the impact of the test result*,* also on life planning*
* In case of positive genetic result: discuss implications of the test result for participation in clinical trials*,* ask for permission to share genetic results with research team*
* Offer to refer participants to the relevant medical professional for psychological counselling*
* Provide information on post-disclosure follow-up*
**Stage 4: Post-disclosure follow-up with FTD researcher**
Conduct follow-up phone calls or video calls with the participant and their family members, depending on participants’ and families’ needs, but at least once after the first week. Assess well-being and processing of the result, test comprehension of the result, answer any questions, and create an appropriate follow-up plan (e.g., referral to psychologist). Be alert to serious psychological impact of the OPBT result.
In case of positive OPBT result *with positive genetic result*: arrange follow-up for FTD symptoms, frequency of check-ups can be decided by participant and researcher
In case of positive OPBT result *with positive genetic result*: discuss next steps for participation in clinical trial, emphasize that willingness to participate has no consequences for access to or quality of follow-up care
In case of positive OPBT result *with positive genetic result*: ask whether the participant wants the test result to be communicated to the primary care physician and/or documented in their medical record

Table caption: Italics: only discussed with individuals at 50% risk, underlined: only discussed with known carriers

**Table 2:** Discussion points for the first-time pre-test counselling session and psychological consultation

Discussion point	Key content and questions
Information on the testing procedure	• Modality (blood/MRI/lumbar puncture) and other details of sample collection, e.g., how many samples are collected at what time points
Information on meaning and predictive value of test result	• What the biomarker is (e.g., breakdown product of neurons)
	• (Gene-specific) time to onset with precision range, and what is meant by ‘onset’
	• Cut-off values, positive and negative predictive value, complexity of interpretation of results, potential need for re-testing
	• Potential alternative explanations of positive result
	• Remaining uncertainty of progression rate and type of symptoms
*Implications of positive test result for genetic status (including information usually included in genetic counselling)*	*• With non-specific biomarkers: possibility of positive OPBT result while not a carrier and alternative explanations*
	*• Need for confirmation of positive OPBT result with genetic test *
	*• Process of step-wise disclosure, including waiting times: first OPBT result disclosure, then genetic test in clinical care setting, and finally genetic test result disclosure after several weeks*
	*• Potential implications for risk status of family members and family relationships*
	*• Potential implications for insurance and employability (if applicable in national context)*
	*• Potential implications for reproductive decision-making (if applicable)*
	? *How would your family members react to learning your genetic status and implications for their own status?*
Information about follow-up and clinical trial participation	• Follow-up care after receiving a positive OPBT result *and a positive genetic result* for the counselee and their family members, information about the clinical trial, and process of clinical trial invitation
Reasons for and against receiving test results	? Why do you (not) want to know your OPBT result?
	? *Why does OPBT change your decision to learn your genetic status?*
Intentions for making changes after receiving test results	? What would you do after receiving a negative OPBT result?
	? What would you do after receiving a positive OPBT result *with a positive genetic result*? (in domains of work, activities, life planning, advance care directives, euthanasia directives, insurance, other)
Expectations of psychological impact and coping	? Which result do you expect to receive?
	? What are the potential psychological impacts you could experience when receiving a positive OPBT result? And your family members?
	? How do you think you will cope with a positive OPBT result, and what care resources do you think you will need?
Support network and sharing results	? Who might support you in coping with the test results?
	? With whom would you share the test result and when?
	? How would a positive OPBT result affect your self-image and social interactions with others?

Table caption: Italics: only discussed with individuals at 50% risk. ● : talking point. ? : recommended question

### Part 2: Condensed process for repeated disclosure of OPBT results

With each repetition of OPBT or OPBT-based eligibility screening for a clinical trial, participants should be recommended to go through the condensed disclosure process to revisit the most important information about OPBT disclosure.

The condensed process contains a stage 0, in which participants are informed about repeated OPBT and are sent a written summary of all relevant information regarding OPBT before pre-test counselling, including information on the clinical trial for which their eligibility will be assessed. Stage 2 to 4 are the same as in the first-time disclosure process, but time to consider between pre-test counselling and testing may be shorter than with first-time disclosure. Stage 1 discussion points are the same as stated in Table 2, but should be recapped and adapted to fit the previous experience and knowledge level of participants with OPBT disclosure, based on the documentation of the participant’s previous considerations (if available, see Part 1). It should be emphasized that participants are free to discontinue or postpone OPBT disclosure. Especially if participants are experiencing or have experienced serious psychological impacts from previous experiences with OPBT, the physician-researcher and participant should discuss whether to undergo OPBT at the current time. Similar to first-time disclosure, psychological support can be offered or requested, for instance if a family member has recently developed FTD or passed away from it, if there have been significant life events or changes in living circumstances or in case of increased test-related distress. If considerable time has passed since the participant received pre-test counselling for OPBT disclosure, counsellors should consider counselling participants following the extensive process in Part 1. Table 3 provides an overview of stages 0 and 1 in the condensed process.

**Table 3:** Stages of the condensed process for known carriers and individuals at 50% risk of genetic FTD

**Stage 0: Education material prior to pre-test counselling**
Send the written summary of relevant information on OPBT to participants for rereading prior to counselling
**Stage 1: Pre-test counselling session with FTD physician-researcher**
Recap information in Table 2
Refer back to previous pre-test counselling discussions and ask whether the participant has new considerations regarding willingness to learn OPBT results
Stress that OPBT is offered to assess eligibility for a clinical trial of an investigational treatment of unknown efficacy, and participants may discontinue or postpone OPBT at any time
Provide information about the possibilities for participation in a clinical trial in case of a positive result, and describe potential risks and benefits of participation in the trial
Offer additional sessions with a clinical geneticist, medical psychologist or other relevant professionals if participant wishes
Check whether preferences for the disclosure session (time, mode) are the same
Discuss process and preferences for follow-up and/or clinical trial invitation (if applicable) after disclosure

## Discussion

This disclosure process aims to guide FTD researchers in the ethically responsible implementation of OPBT disclosure to individuals at risk for genetic FTD in the context of clinical trial recruitment. Most elements of the framework are based on previous disclosure processes developed for genetic and biomarker results in HD [11, 12], ALS [13, 14] and AD [15–21] (see Supplement 1), but have been slightly adapted to fit the context of FTD research. We also took into account recent empirical findings on the perspectives and preferences of individuals at risk of genetic FTD regarding OPBT disclosure [22]. We recommend that the process be followed closely for all participants, as participants in a community with a rare genetic disease may share their experiences with each other and any discrepancies in the process could lead to speculation about the test results.

The process was designed both for eligibility screening for specific clinical trials and for biomarker-based monitoring of ‘trial readiness’ in the context of longitudinal cohort studies. However, the order in which counselling should be offered will differ across these two settings. If individuals are invited to undergo OPBTs as part of eligibility screening for a specific clinical trial, they should only be invited to do so if they meet al.l other eligibility criteria for that specific trial in order to avoid disclosure of positive OPBT results to individuals who turn out to be ineligible for that trial based on other criteria. Moreover, potential participants should receive all relevant information about that specific trial prior to deciding about their willingness to undergo eligibility screening. The information should include potential risks and benefits and any consequences of participation for their future eligibility for other clinical trials. In contrast, in ‘trial readiness’ cohorts, counselling for OPBT monitoring will take place at longitudinal study enrolment before specific clinical trials are in view. Consent should then be asked for both monitoring and recontact for participation in a clinical trial based on monitoring results [24]. This consent should be renewed at each longitudinal study visit using the condensed process. If participants meet eligibility criteria for a specific clinical trial and are invited, the physician should give them the option not to proceed with disclosure of monitoring results and clinical trial participation.

Although OPBT are being used for clinical trial recruitment, they are currently not sufficiently predictive and validated to be implemented in clinical care, nor are they routinely returned as individual research results in longitudinal cohort studies. As the development of OPBTs progresses, the process should be updated to keep pace with changing needs of researchers and clinicians who are disclosing OPBT results to individuals at risk of FTD.

As this article presents a general framework for disclosure of OPBT results, details need to be specified before the process can be put into practice. Firstly, the process does not concentrate on a specific OPBT, but the type of biomarker used may influence the process significantly. For example, if a biomarker is dynamic and fluctuates over time (as for example NfL does), multiple testing moments may be required to establish a consistently positive or negative result before results can be disclosed. Alternatively, the clinical trial may have inclusion criteria that are based on other types of results besides “positive” or “negative” (e.g. intermediate results), necessitating adaptation of pre-test information provision about possible outcomes. If results are in the grey zone between positive and negative, this may also require further counselling.

Secondly, the population invited to undergo OPBT may differ per study site, which may influence the execution of the disclosure process. Most trial participants will probably be recruited from existing longitudinal cohort studies, such as GENFI. In these studies, samples that are collected routinely for research purposes may be used for OPBT for the purposes of trial recruitment, and so sample collection (stage 2: test procedure) may already have taken place. In addition, researchers recruiting from longitudinal cohort studies are more likely to already be acquainted with the participant, their family context and prior decision-making regarding genetic testing or OPBT. This will facilitate tailoring pre-test counselling to the individual. Still, prior acquaintance with the participant is not a prerequisite for proper execution of the disclosure process. If the counsellor is not already acquainted with the participant, they may take more time to get to know the participant in pre-test counselling besides covering talking points in Table 2.

Thirdly, the process will need to be adapted to the national, local and cultural context of the study site. For example, a larger geographical distance from participants to the genetic counsellor or limited availability of trained counsellors may limit opportunities for or the desirability of in-person counselling. In that case, pre-test counselling may be conducted via videocall instead. Another example is that some legal contexts allow insurance companies to use clinical genetic test results, disclosed to participants at 50% risk during stage 3b, for underwriting purposes. In those contexts, participants may prefer to receive genetic test results (stage 3b) in the research context rather than being referred for clinical genetics services. Overall, we have tried to design a disclosure process that is as broadly applicable as possible, at least within Europe, but some elements may need to be adapted to make its implementation at a specific study site feasible.

There are a few open questions that need to be highlighted when considering the disclosure of OPBT results to individuals at risk of genetic FTD. First, although we have included guidance for disclosure of OPBT results to individuals at 50% risk, there is currently no consensus regarding the ethical acceptability of disclosing onset predictions to people who are unaware of their genetic status [10]. On the one hand, individuals at 50% risk seem willing to learn their genetic status if this is coupled with a prediction of onset in the coming years and if it makes them eligible for clinical trial participation [22]. In addition, individuals at 50% risk make up the majority of the population that participate in longitudinal cohort studies of FTD. Their inclusion in eligibility screening using OPBT would expand the pool of potential participants for clinical trial participation, increasing the feasibility of successfully conducting clinical trials for this rare disease. On the other hand, one reason for individuals at 50% risk of genetic FTD not to undergo genetic testing is fear of experiencing negative psychological impacts from receiving a positive result [25, 26]. If they learn simultaneously that they are carriers of the genetic mutation and that they are expected to develop symptoms in the next few years, they may be more heavily affected than carriers who have been aware of their genetic status for a longer period of time [10]. The GENFI Participant Engagement Board emphasized the potential for serious negative psychological impact of positive OPBT results, including suicide, and the need for high-quality counselling to protect participants and their family members from harm. In the absence of evidence on the impact of OPBT, researchers will need to decide whether to include individuals at 50% risk in OPBT protocols or not. If they do, this disclosure process offers guidance to provide comprehensive counselling, step-wise consideration of willingness to continue and psychosocial support in the challenges of counselling individuals at 50% risk and their family members.

A second open question concerns the level of understanding that can be expected of participants when counselling them about possible outcomes, risks and benefits of OPBTs. There are several challenges: some OPBTs may be based on non-specific biomarkers such as NfL, complicating the interpretation even of a positive result. In addition, a repetition of the test over time may be necessary to increase predictive value [27], but the interpretation of inconsistent results may be complicated. Also, OPBT results will probably consist of an estimate of the time to onset, an estimate of the range of precision of this estimate, and possibly metrics like positive and negative predictive value. All this information is probabilistic and complex, requiring caution and clarity when explaining test features in pre-test counselling. It must be made as clear as possible what information OPBTs can and cannot provide. In longitudinal cohort studies, the researcher may be familiar with the participant (and their family), allowing them to tailor the information and considerations discussed with the participant to improve their understanding of the information. Yet, even with the use of visual resources such as pictographs, it will be challenging to make sure that participants understand all relevant information. The question then becomes what level of understanding is sufficient to meet requirements for informed consent.

A third open – ethical – question is raised by the direct link between some participants’ willingness to receive OPBT results and the opportunity to participate in a clinical trial. Carriers and individuals at 50% risk of genetic FTD may see clinical trial participation as their only hope to delay the disease process or contribute to a better future for their at-risk children. They are usually eager to participate for their own or the community’s interests in new therapeutic options. There have been reports of individuals who pursued genetic testing in order to be eligible for participation in clinical trials or “get on the list” [Graafland et al. unpublished results]. The inclusion criterion of testing positive on OPBTs for clinical trials may exert a similar influence on participants to accept OPBT disclosure, even if other considerations might favour refusing OPBT. As Kim et al. point out, this may not amount to coercion, since clinical trial participation is not a right and OPBT disclosure is simply a condition for participation [28]. Still, counsellors should be aware that a participant’s motivation to participate in a clinical trial may influence their willingness to accept OPBT disclosure and should discuss this during pre-test counselling. They should also make sure that the motivation to participate in a clinical trial is not influenced by therapeutic misconceptions.

Fourth, lack of a shared understanding of what ‘disease onset’ means in pre-test counselling raises ethical concerns. There is a difference between biological onset, which refers to the development of biological pathology as measured by biomarkers, and clinical onset, which refers to the moment at which the individual develops symptoms of FTD. The difference between these two conceptions of onset may not always be comprehensible for individuals at risk of genetic FTD. Furthermore, clinical onset is often difficult to establish, and diagnostic delay often occurs due to the clinical heterogeneity of FTD, the subtlety of first symptoms, and misdiagnosis as a psychiatric disease [29, 30]. There have been recent efforts to define a prodromal phase of FTD, in which symptoms are already present but the affected individual does not yet meet diagnostic criteria [31, 32], similar to efforts to define mild cognitive impairment in AD. During the prodromal phase, the receipt of abnormal onset-predictive biomarker test results entails a risk of hypervigilance, turning normal cognitive lapses or changes in behaviour into perceived symptoms [3, 22]. Overall, clinicians and researchers developing OPBTs should be aware that the word ‘onset’ may have different meanings for different individuals. They need to make clear what definition of disease onset they use [33] as an explicit topic of discussion during pre-test counselling.

Finally, an important knowledge gap is the current lack of insight into the psychological and social impacts of disclosing OPBT results in genetic FTD. This should be a topic of investigation once disclosure processes are implemented by monitoring the impact of disclosure on research participants in the context of clinical trial recruitment. In a previous article, we recommended disclosing OPBT results to a small group of individuals at risk of FTD first and monitoring their reactions and those of their families in order to inform any further disclosure processes in larger groups of participants [10]. A previous interview study on expected effects of disclosure of OPBT results suggests that disclosure could have effects on mood, hypervigilance, surveillance, self-image (even a nocebo effect), behaviours or life choices [22]. A mixed-methods approach combining neuropsychological measures, psychological measures (e.g., the Warwick-Edinburgh Mental Wellbeing Scale, the Hospital Anxiety and Depression Scale, the Illness Identity Scale, the Psychological Adaptation Scale and/or the Impact of Events Intrusion and Avoidance Subscale) and qualitative methods to explore lived experiences after disclosure of OPBT results could provide valuable information to inform pre-test counselling and follow-up needs for the larger population.

## Conclusions

There is an urgent need to prepare for disclosure of onset-predictive biomarker results in the FTD research context, as these biomarkers are set to be used in clinical trial recruitment. The first steps into this new territory of disclosing OPBT results to individuals at risk of genetic FTD are challenging and unpredictable, but if done well, the information may be very beneficial to these individuals. Hopefully, the outlined disclosure process with pre-test counselling and high-quality follow-up will contribute to a responsible disclosure practice in the context of clinical trial recruitment and other contexts in the future.

## Supplementary Information

Supplementary Material 1.

## Data Availability

No datasets were generated or analysed during the current study.

## Abbreviations

AD: Alzheimer’s disease
ALLFTD: ARTFL LEFFTDS Longitudinal Frontotemporal Dementia study
ALS: Amyotrophic lateral sclerosis
APOE: Apolipoprotein E
CDR®: Clinical Dementia Rating Dementia Staging Instrument
CDR® plus NACC FTLD: Clinical Dementia Rating Dementia Staging Instrument plus
NACC FTLD: Behavior & Language Domains
FTD: Frontotemporal dementia
GENFI: Genetic Frontotemporal Dementia Initiative
HD: Huntington’s disease
NfL: Neurofilament light chain
OPBT: Onset-predictive biomarker test
TDP-43: TAR DNA-binding protein-43
