# Supplementary material for: Disclosure of onset-predictive biomarker results to research participants at risk of genetic frontotemporal dementia: a European perspective
Source: Alzheimers Res Ther. 2025 Dec 13;18:15. doi: 10.1186/s13195-025-01930-4 (PMC12817822; doi:10.1186/s13195-025-01930-4)
Supplement: Supplementary file 1 — Supplementary Material 1. [file 13195_2025_1930_MOESM1_ESM.docx]

Supplement 1. Steps and actions in the disclosure process that were taken from recommended processes of genetic and biomarker disclosure in HD, ALS and AD.

| **Steps** | **Taken from** |
| --- | --- |
| Structure disclosure as pre-test counselling, testing, disclosure and follow-up | 11-13, 16, 20 |
| Give time to consider after pre-test counselling | 11-13, 20 |
| Recommend to bring a support person | 11-13, 20 |
| Use clinical judgement to decide whether to disclose OPBT results in case of psychiatric problems | 11, 20 |
| Send a written summary of information | 11-21 |
| Talking points for pre-test counselling | 11-21 |
| Inform participants of the right to withdraw or postpone disclosure | 11-13, 16, 17 |
| Tailor follow-up | 11, 12, 14, 16, 20 |
| Provide information on clinical trials | 11-13, 17, 20 |
| Train counsellors in (study-specific) counselling methods | 12, 14, 16, 17, 20, 21 |
| Ask whether to communicate results to primary care physician or put in medical record | 12 |
| Provide counselling, disclosure or follow-up via videocall or telephone if preferred | 13, 15, 17, 21 |
| Only disclose OPBT results if otherwise eligible for trial participation | 14 |
| Use condensed process | 17, 19 |
| Assess psychological impact of disclosure | 17, 20, 21 |

References:

1. Huntington’s Disease Society of America (HDSA). Genetic testing protocol for Huntington’s Disease. New York: HDSA, 2016.
2. MacLeod R, Tibben A, Frontali M et al. (2013) Recommendations for the predictive genetic test in Huntington's disease. Clin Genet 83(3):221-231. https://doi.org/10.1111/j.1399-0004.2012.01900.x
3. Benatar M, Stanislaw C, Reyes E, et al. (2016) Presymptomatic ALS genetic counseling and testing: Experience and recommendations. Neurology 86(24):2295-2302. https://doi.org/10.1212/WNL.0000000000002773
4. Benatar M, Wuu J, Andersen PM, et al. (2022) Design of a Randomized, Placebo-Controlled, Phase 3 Trial of Tofersen Initiated in Clinically Presymptomatic SOD1 Variant Carriers: the ATLAS Study. Neurotherapeutics 19(4):1248-1258. https://doi.org/10.1007/s13311-022-01237-4
5. Christensen KD, Uhlmann WR, Roberts JS, et al. (2018) A randomized controlled trial of disclosing genetic risk information for Alzheimer disease via telephone. Genet Med 20(1):132-141. https://doi.org/10.1038/gim.2017.103
6. Harkins K, Sankar P, Sperling R, et al. (2015) Development of a process to disclose amyloid imaging results to cognitively normal older adult research participants. Alzheimers Res Ther 7(1):26. https://doi.org/10.1186/s13195-015-0112-7
7. Langlois CM, Bradbury A, Wood EM, et al. (2019) Alzheimer's Prevention Initiative Generation Program: Development of an APOE genetic counseling and disclosure process in the context of clinical trials. Alzheimers Dement 5:705-716. https://doi.org/10.1016/j.trci.2019.09.013
8. Lingler JH, Butters MA, Gentry AL, et al. (2016) Development of a Standardized Approach to Disclosing Amyloid Imaging Research Results in Mild Cognitive Impairment. J Alzheimers Dis 52(1):17-24. https://doi.org/10.3233/JAD-150985
9. Roberts JS, Chen CA, Uhlmann WR, Green RC (2012) Effectiveness of a condensed protocol for disclosing APOE genotype and providing risk education for Alzheimer disease. Genet Med 14(8):742-748. https://doi.org/10.1038/gim.2012.37
10. Largent EA, Grill JD, O'Brien K, Wolk D, Harkins K, Karlawish J (2023) Testing for Alzheimer Disease Biomarkers and Disclosing Results Across the Disease Continuum. Neurology 100(21):1010-1019. https://doi.org/10.1212/WNL.0000000000206891
11. Robillard JM, Masellis M, Martin SE, Khachaturian AS, Dixon RA (2024) The Return of Biomarker Results in Research: Balancing Complexity, Precision, and Ethical Responsibility. J Alzheimers Dis 97(3):1083-1090. https://doi.org/10.3233/JAD-230359
